# Policy implementation and financial incentives for nurses in South Africa: a case study on the occupation-specific dispensation

**DOI:** 10.3402/gha.v6i0.19289

**Published:** 2013-01-24

**Authors:** Prudence Ditlopo, Duane Blaauw, Laetitia C. Rispel, Steve Thomas, Posy Bidwell

**Affiliations:** 1 Centre for Health Policy, School of Public Health, Faculty of Health Sciences, University of the Witwatersrand Johannesburg, South Africa; 2 School of Public Health, Faculty of Health Sciences, University of the Witwatersrand, Johannesburg, South Africa; 3 Centre for Health Policy and Management, Trinity College, Dublin, Ireland

**Keywords:** financial incentives, occupation specific dispensation, nurses, policy implementation, South Africa

## Abstract

**Background:**

In 2007, the South African government introduced the occupation-specific dispensation (OSD), a financial incentive strategy, to attract, motivate, and retain health professionals in the public sector. Implementation commenced with the nursing sector, but there have been unintended negative consequences.

**Objective:**

First, to examine implementation of the OSD for nurses using Hogwood and Gunn's framework that outlines ‘perfect implementation’ pre-conditions. Second, to highlight the conditions for the successful implementation of financial incentives.

**Methods:**

A qualitative case study design using a combination of a document review and in-depth interviews with 42 key informants.

**Results:**

The study found that there were several implementation weaknesses. Only a few of the pre-conditions were met for OSD policy implementation. The information systems required for successful policy implementation, such as the public sector human resource data base and the South African Nursing Council register of specialised nurses were incomplete and inaccurate, thus undermining the process. Insufficient attention was paid to time and resources, dependency relationships, task specification, and communication and coordination.

**Conclusion:**

The implementation of financial incentives requires careful planning and management in order to avoid loss of morale and staff grievances.

Financial incentives are a commonly used strategy to improve health worker motivation and retention (1, 2). However, there is limited evidence of their impact in low- and middle-income countries (3, 4) or in rural and remote areas (1, 3). Rigoli and Dussault have cautioned that the failure of many incentive schemes to achieve expected results is due to poor design (5). The problems of inappropriate or poorly designed policies are exacerbated by policy implementation challenges, which in turn may determine the impact of financial incentive policies (6, 7). In most countries, policy makers have limited guidance on how to implement financial incentive strategies so that they can achieve their intended policy objectives. For example, inequities, or perceived inequities in the manner in which incentive strategies are designed and implemented have been documented as a source of demotivation (4). Increasing attention has thus been paid to understanding the problems of policy implementation in order to identify key contributing factors to the failure to achieve the desired impact (8).

In South Africa, several authors have analysed the disjuncture between policies or plans, on the one hand, and implementation, on the other hand. These have included studies on mental health (9–11), HIV and AIDS (12–15), hospital rationalisation and restructuring (12, 14), municipal health services (16), social inclusion (17), and user fees (18). A few studies have looked at policies on remuneration or incentives (19–21). Several problems have been identified. Stack and Hlela, and Khosa found that the challenges included hurried policy implementation; limited consultation with the implementing actors; lack of prioritisation, insufficient time, and a lack of co-ordination between different government departments (12, 14). Rispel et al. found that inadequate administrative and implementation capacity, insufficient resources, corruption, and lack of involvement of civil servants beset the implementation of various social policies (17), while challenges of varying technical capacity at different government levels influenced the implementation of mental health policy (11).

Although these studies provide important insights into the factors that plague policy implementation, there is a dearth of studies that analyse the implementation of policies on remuneration or financial incentives (19–21). In light of the global human resource crisis, analysis of policy implementation of financial incentives is important to guide policy makers, as these are the most commonly used staff retention strategies.

In South Africa, public sector conditions of service (issues such as remuneration or working hours) are determined on the basis of a central collective bargaining process between the employer (government) and recognised labour representatives (unions or professional associations), in line with provisions of the Labour Relations Act (22).

In 2007, the South African government introduced the occupation-specific dispensation (OSD), a financial incentive strategy to attract, motivate, and retain health professionals in the public health sector (23). The OSD policy defines the remuneration structure, frequency of pay progression, grade progression opportunities, career pathing, recognition of appropriate experience and required levels of performance (23, 24). Implementation commenced with all categories of nurses, as they are the majority of health care providers in South Africa and because of the shortages of and high turnover among nurses in clinical specialities, especially in critical care wards and operating theatres.

Existing evidence suggests numerous problems with the implementation of the OSD policy, ranging from inadequate planning, budget overruns (25) and some unintended negative consequences (26). These consequences included unmet nurses’ expectations, inequities in the amounts received, perceived unfairness, and dissatisfaction and divisions among the different categories of nurses (26).

The article draws on the Hogwood and Gunn policy implementation framework to analyse the implementation of OSD and seeks to determine whether the manner in which OSD was implemented caused these unintended negative consequences. The article concludes with lessons for South Africa and other countries, which could be used to guide future financial incentive policies.

## Conceptual framework

Although there are several theories and frameworks to analyse policy implementation, this article uses Hogwood and Gunn as an analytical framework (Table 1) to analyse the implementation of OSD (7, 27). Hogwood and Gunn make a distinction between ‘non-implementation’ and ‘unsuccessful implementation’. With ‘non-implementation’, policies are not put into effect as intended because those involved in the implementation are inefficient or because it was not possible to overcome obstacles despite their best efforts. ‘Unsuccessful implementation’ refers to a situation where the policy is fully implemented but fails to produce the intended outcomes (7). Although the framework is idealistic in that it suggests the notion of ‘perfect’ implementation, it is useful to consider the preconditions and key features necessary for the successful policy implementation of complex financial incentives.

**Table 1:** Conceptual framework

Hogwood and Gunn's 10 preconditions for implementation (27)	

Stage	Description of the practice
External constraints	Circumstances external to the implementing agency do not impose crippling constraints
Time and resources	Adequate time and sufficient resources are made available to the programme
Resource combination	The required combination of resources is available
Theory-based policy	The policy to be implemented is based on a valid theory of cause and effect
Cause/effect relations	The relationship between cause and effect is direct and that there are few, if any intervening links
Dependency relationships	The dependency relationships are minimal
Agreements of objectives	There is agreement of, and understanding on, objectives
Events sequencing	The tasks are fully specified in correct sequence
Communication/coordination	There is perfect communication and coordination
Total compliance	Those in authority can demand and obtain perfect compliance

## Methods

The study was approved by the University of the Witwatersrand Committee for Research on Human Subjects and the Provincial Health Research Ethics Committees in the two provinces that participated in the study. Hospital managers were also asked for permission to access their facilities. All participants provided informed consent after receiving a study information sheet.

### Study design

A descriptive case study design was used, combining a review of policy documents and in-depth interviews with key informants who were knowledgeable on OSD policy design and/or implementation. The document review included relevant government documents including policy directives (23, 24) and media releases (28–30) in order to understand the context and content of the OSD implementation.

### Study sites and setting

The study was conducted in North West and Gauteng provinces between 2008 and 2010. North West is a predominantly rural province with a population of 3.2 million (6.4% of the total South African population) while Gauteng is an urban province with a population of 11.1 million (22.4% of the total population). We selected a total of 10 hospitals in the two provinces. Out of 30 hospitals in North West province, three were purposively selected^1^ to ensure inclusion of those hospitals that were part of the hospital revitalisation programme and two were selected randomly. Similarly, in Gauteng province, one hospital was purposively selected while four were selected at random.

### Study participants and data collection

Forty-two key informants were selected purposively on the basis of their influence or knowledge of the OSD policy or their involvement with OSD implementation, using a snowballing sampling technique. The key informants consisted of the following groups of stakeholders: national government (7), provincial government (*n*=12), academics (*n*=7), statutory body (*n*=1), nursing organisations (*n*=4), nursing unions (*n*=3), and hospital managers who were available at their facilities on the day of data collection (*n*=8).

The selected key informants were interviewed using a semi-structured interview guide. The questions focused on the background and context of OSD for nurses, actors and their responsibilities, implementation of OSD, successes of the OSD implementation, challenges experienced, and recommendations.

### Data analysis

A thematic content analysis of the documents and transcripts was conducted (31) using ATLAS.ti. To ensure coding consistency, two researchers (one being the first author) independently read at least seven transcripts and discussed discrepancies until agreement on the codes was reached.

## Results

Table 2 shows the extent to which OSD implementation met the preconditions and key features necessary for successful policy implementation, proposed by Hogwood and Gunn. Although these conditions overlap, each is elaborated below for the sake of clarity.

**Table 2:** Assessment of occupation-specific dispensation (OSD) implementation against Hogwood and Gunn conditions for success

Hogwood and Gunn's criteria	OSD assessment
External constraints	Widespread public sector strike accelerated implementation, contributing to insufficient planning before implementation Some contestation over prioritisation of nurses as the first health provider category to benefit from OSD
Adequate time and resources	OSD implementation was rushed Insufficient financial resources Limited human resources Inadequate training of the implementers
Required combination of resources	OSD policy guideline in the form of Resolution 3 of 2007 Poor human resource information system
Policy based on valid theory	Philosophy of OSD policy supported Policy not evidence based
Clear cause and effect relationship	OSD would provide clear career paths and salary progression for nurses Increase in salary will increase motivation and retention
Minimal dependency relationships	Complex series of events Dependent on nurses submitting proof of Nursing Council qualifications
Agreement of objectives	The objective of OSD was to retain nurses within clinical areas Vagueness in the definition of what constitutes specialisation
Sequencing of events	Roles of implementation stakeholder not made explicit Announcement made prior to ensuring sufficient and combination of resources
Communication and coordination	Weak communication to frontline nurses Poor coordination amongst key actors
Total compliance	Varied interpretation at institution and provincial level Overpayments and underpayments

### Precondition 1: external constraints

External obstacles ‘may be political in that either policy or the measures needed to achieve it are unacceptable to interests (such as party activists, trade unions, or in some societies, the military) which have the power to veto them’(27).

Although OSD was part of ongoing government efforts to recruit and retain health care professionals in the public sector, intense lobbying by organised labour, particularly by the Democratic Nursing Organisation of South Africa (DENOSA), for improved remuneration and a protracted public servants strike during 2007 accelerated its implementation (32). However, the strike influenced the ability of government to plan adequately.

A key external constraint was contestations regarding the prioritisation of nurses over other health care professionals in the OSD implementation, especially among the recognised labour organisations in the central bargaining chamber. One informant commented as follows:

At that time, government said I have got X amount of money to improve the salaries of health professionals, but it is not enough to improve the salaries of everybody. Now all of us as health professionals had to say ‘start with us’ so it was so difficult. So when we thought that we had convinced the government to start with the nurses, when it went to the Bargaining Chamber with other trade unions, we were seen as people that were promoting elitism. We were asked ‘why only nurses, what about cleaners?’ (KII 18, Nursing Union)

### Preconditions 2 and 3: adequate time and resources

Both Gauteng and North West provinces reported using the OSD policy for implementation. Task teams were established at some hospitals, and these hospital managers reported fewer implementation challenges.

One of the reasons for the success of the OSD process was the formation of the task teams prior to implementation. (Assistant Nursing Manager, Central Hospital, Gauteng)

A combination of resources is required according to the Hogwood and Gunn framework, which assumes that if one or more of the resources is delayed, the project is set back by several months (27). Although the intention was for OSD to be implemented by March 2008 (23), actual implementation was delayed until September 2008; and even then, not all provinces implemented it at the same time. There were inconsistencies in the benefits nurses received among the different provinces and institutions. This led to confusion and disgruntlement.

Insufficient time was allocated for the training of the implementers at institution level, there was no reference made in the OSD policy to who would be trained on OSD implementation or the length of the training (23), so this aspect was neglected. Thus reinforcing, a common reason why policies do not achieve the stated intentions is that too much is expected too soon (27). There was consensus amongst hospital managers that OSD was implemented in a rushed and pressured manner.

I think the way OSD was implemented, it was in a rush, there were deadlines to say on a certain date implementation must start. It was putting a lot of people under pressure. I think it would have been done smarter if it was done at a natural pace and everybody has been comfortable with the implementation. (Hospital Manager 4, District Hospital, Gauteng Province)

In terms of financial resources, around R1, 5 billion (equivalent to US$ 200 million; 1$=7.5 South African Rands) was allocated for OSD for nurses for the 2007 financial year (Department of Health, 2007). This amount was insufficient for several reasons. Firstly, a sub-optimal human resource information system resulted in an under-count of the total number of nurses in the public health sector by 10,000 individuals. Although this was a policy design problem, it had ramifications during implementation. Hence, the implementation of the OSD proved to be more expensive than previously planned, illustrated by the comment from one key informant.

It became clear that the government doesn't know how many nurses they actually have. I remember they were even roughly quoting figures, only to find that they under-budgeted around 10,000 nurses. (KII 18, Nursing Union)

A second key challenge was that financial resources were not made available immediately at the hospital level, resulting in problems for individual hospital managers.

The people who planned it [OSD] are wrong because you can't plan to give people money whilst you don't have that money at hand. In our case, we had to pay the nurses even though we didn't budget for OSD. It was said that the budget for OSD will come from the province, but it never came at that time. (Hospital Manager 9, District Hospital, North West Province)

Lastly, insufficient financial resources were exacerbated by problems of overpayment of some nurses, and this meant that hospitals could not fill vacant posts.

People were paid more than they were supposed to be paid, so that's why now we can't even fill vacant posts. (Hospital Manager 9, District Hospital, North West)

### Preconditions 4 and 5: valid theory, clear cause, and effect relationship

The study found that Hogwood and Gunn's preconditions 4 and 5 were met. There was general agreement amongst respondents that OSD was a ‘good’ policy, which was informed by the human resource problems in the public health sector, illustrated by the following comments:

Well, in theory it [OSD] was a brilliant idea and it should have gone a long way to solve some of the problems. (KII 7, Gauteng Department of Health)

The nurses’ OSD remains the best possible policy to address the issues within nursing. (KII 20, Nursing Union)

The OSD also attracted nurses from overseas back into the country and from the private to the public health sector.

But I have realised that when I was in PE, just before I came here, the introduction of the OSD attracted a lot nurses from the private sector … when I came here in 2008 February, we employed about 160 nurses from the private sector. (KII 10, Eastern Cape Department of Health)

… turnover was high … there was a time when we were really struggling to recruit nurses, but I must say the OSD made a huge difference because we are able to recruit people from private sector and we also got two nurses from overseas. (Hospital Manager 4, District Hospital, Gauteng Province)

Key informants were also of the opinion that OSD encouraged nurses to improve their qualification or to specialise in critical care or operating theatre technique.

Nevertheless, some respondents pointed out that OSD was not based on research evidence and were of the opinion that formative research should have been conducted to shape this policy.

I think before they could implement it [OSD], they were supposed to do thorough research and correct calculations. (Hospital Manager 9, District Hospital)

For precondition 5, it can be argued that the causal chain was direct (7). This is because there was an expectation from policy makers that an increase in salary would increase recruitment and retention of nurses.

### Precondition 6: minimal dependency relationships

Minimal dependency relationships imply that there should be a single implementing agency or if other agencies are involved, that the dependency relationships are minimal both in number and importance (27). However, in terms of the South Africa constitution, health is a concurrent function (or joint responsibility) of the national and provincial governments, with national government responsible for overall policy development, while the nine provincial health departments are the implementing agencies. Invariably, this decentralisation resulted in different interpretations and variations in policy implementation.

OSD implementation was also dependent indirectly on other agencies such as the South African Nursing Council (SANC), as nurses were required to submit proof of their registration and specialised qualifications with the SANC. Nurses were also required to produce their length of service record. Consequently, an informant from a nursing union blamed the SANC for failure to record the relevant nursing qualifications.

I must say SANC nearly failed the [nurses] on OSD, there are things in SANC that we must correct, around the registration of certain courses, accreditation of courses and so forth because we have a large chunk of nurses who were left out of the OSD because they are not regarded as speciality. (KII 20, Nursing Union)

On the other hand, SANC shifted the blame on the nurses for their tardiness in registering their qualifications and only doing so when these were required as part of the OSD implementation.

### Precondition 7: agreement of objectives

The document review suggests that the policy makers did not take into account the complexities of the various nursing specialities. One respondent remarked as follows:

Nursing is a complicated profession, there are different specialities. So nursing OSD is not as straight forward as any OSD. I think there were grey areas in the implementation of OSD or no clarity (KII 27, Nursing Manager)

For example, the OSD policy lacked clarity on how nurses with more than one speciality were to be rewarded. Furthermore, a grandfather clause was introduced that allowed nurses who worked in a speciality area, but without the formal qualification, to benefit from OSD. On the other hand, those with the formal qualification, but not working in the specialised area could not benefit. This resulted in unhappiness among those nurses and movement of some nurses out of managerial positions back to the specialised area, where OSD was paid. A document review of a letter by the Principal Specialist and Head of one Gauteng hospital to SANC chairperson noted some of these concerns:

Fewer registered nurses are choosing to work in medical wards following the implementation of the Occupational Specific Dispensation (OSD). This is because medical nurses do not qualify for OSD as there is not a recognised post-basic qualification in medical nursing. Consequently, there are fewer registered nurses in medical wards and care of medical inpatients is severely compromised. (Principal Specialist and Head, Regional Hospital, Gauteng Province)

### Precondition 8: sequencing of events

The announcement on the OSD was made by the then Minister of Health, Dr Manto Tshabalala-Msimang prior to ensuring that sufficient financial and human resources were in place. This on its own created a lot of confusion and dismay among nurses when the implementation was delayed at provincial level.

Another important element of sequencing is that the role of each stakeholder involved in the implementation process should be made explicit (27). The OSD policy did not state the responsibilities of provincial or hospital managers explicitly nor the steps required for effective implementation. This resulted in variations in implementation across the nine provinces. One hospital manager commented as follows:

Provinces did as they wish, that's why a nurse in here can be paid differently from a nurse in Eastern Cape or a nurse in Limpopo province. For me, the current system does not work because provinces are independent entities; national [government] usually can say here is a guideline and provinces can decide on how they implement it. (Hospital Manager 7, Provincial Hospital, North West Province)

In the study provinces, the OSD implementation was the responsibility of the Human Resource divisions of the provincial health departments. However, they were not familiar with nursing training and specialities. This challenge was exacerbated by the unavailability of uniform instructions and inadequate training to support these managers. It was left to these human resource managers to define their role, liaise with the hospital or provincial managers and fulfil these undefined roles to the best of their abilities.

### Precondition 9: communication and coordination

The OSD policy clearly stipulated that its fundamental underpinnings are ‘career pathing’, ‘pay and grade progression’, ‘specialty’ as well as ‘competencies’ and ‘performance’. For instance, one of the objectives noted in the policy reads as follows:

To provide adequate and clear salary progression and career pathing opportunities based on competencies, experience and performance. (OSD Policy, Resolution 3 of 2007)

However, there were many mixed messages sent out to nurses, who either received information from the media or their labour organisations. In the media, OSD was promoted as a general nurses’ salary increase and one informant commented as follows:

Unfortunately in the eyes of the nurses, it [OSD] was more about the salary increment. Nurses look at their salary and say ‘no, but this thing [OSD] did not benefit me’. (KII 2, Nursing Union)

Hence, the communication did not emphasise the fundamentals of OSD, resulting in disappointment and unrealistic expectations among nurses. Communication and coordination between the National Department of Health and provinces were also inadequate, and it was not clear what support was given to provinces during implementation or how the provinces in turn supported the districts and hospitals. It was also not clear whether and how coordination happened between the SANC and the provinces or health facilities.

### Precondition 10: total compliance

This precondition suggests that those in authority can demand and obtain perfect compliance. However, the authority for implementation was diffused as the nine provinces and different categories of health facility (primary care centre vs. hospital) interpreted the OSD guidelines differently. Key informants pointed out that in both study provinces, implementation varied across health facilities at the same level, and also between primary health care facilities and hospitals. Inadvertently, OSD contributed to further ‘imbalances’ in the distribution of nurses between hospital and primary health care facilities as well as between provinces.

Lastly, because there was not ‘total compliance’ in terms of policy implementation, accountability was also diffused, and no one took responsibility for implementation challenges. Instead, it became a vicious cycle of blame: the National Department of Health blamed the provinces, the provinces blamed the hospital management, the hospital management blamed the unions, and the unions blamed the SANC, that ironically does not have a mandate for any implementation or participation in financial incentives.

## Discussion

This study used Hogwood and Gunn's 10 preconditions for ‘perfect implementation’ (27) to assess the implementation process of the OSD. Although the framework has been criticised for being idealistic and impossible to achieve (33), it provides useful insights into the challenges of implementing financial incentive policies. The framework is particularly useful in identifying the strengths and weaknesses of the policy implementation process, thus contributing to knowledge on the implementation processes of financial incentives.

The study found that very few of the preconditions were met, namely that the policy was based upon a valid theory of cause and effect and that the relationship between cause and effect is direct (27).

The study found that a number of preconditions were not met, and this resulted in sub-optimal implementation (34). The challenges included time and resources, dependency relationships, task specification, and communication and coordination. For instance, problems with OSD policy implementation that were demonstrated by other policy implementation studies in South Africa (11, 12, 14) included rushed implementation, lack of consultation with or orientation of provincial health departments of health facility managers, lack of proper time scales, and insufficient resources. Because OSD is a financial incentive, with long-term financial implications, these challenges have to be addressed in the planned revision of OSD policy (35).

The implementation of OSD was decentralised, meaning that the responsibility for implementation, including funding was delegated from national to provincial health departments and from the province down to district, sub-district and hospital levels. As was found in other studies (14, 36), this decentralisation resulted in insufficient coordination among different stakeholders and different interpretations and variations in OSD policy implementation. As Jeppson rightly pointed out, policy implementation in a decentralised system is complex and must be understood in a misdirected translation process (37). Consistent with existing policy implementation studies (12, 14, 19, 32), we also found that there was insufficient communication between the different levels of the health departments. Improved communication might have facilitated better implementation (32).

There are several limitations in this study. The use of Hogwood and Gunn's framework limits a detailed analysis of the actors that were involved in policy processes or the identification of intended and unintended impact. This framework further assumes that policy implementation is a linear progression. In practice, this is not the case and it is unlikely that all 10 preconditions of Hogwood and Gunn would be in place prior to implementation of most government policies. Also, the framework does not take into account that setbacks in policy making and implementation are opportunities for revisions, redesign, and self-correction (38). The study was done in two provinces, which limits its generalisability, and the use of key informants reflects their opinions at a point in time.

Nonetheless, there are important lessons to be learned from this case study that could inform the planned revision of the OSD policy. These lessons include the need for better planning and management of the implementation process, careful communication, and change management to ensure that unrealistic expectations are not created, as well as the development of detailed guidelines for implementers and extensive training of these implementers. There is also the need for clear monitoring and evaluation indicators that will allow problems to be picked up at an early stage. This will allow for remedial action to be taken. These lessons are also useful in light of the planned implementation of major health care reforms, notably the National Health Insurance System, thus avoiding a repeat of these mistakes on a much bigger scale.

## Conclusion

The success of financial incentives depends on the manner and process of implementation. Implementation weaknesses can be overcome by paying attention to the conditions needed for successful implementation, namely better planning and management of the implementation process, improved communication and coordination, detailed guidelines for implementers, and clear monitoring and evaluation indicators. However, in acknowledging that ‘perfect implementation’ is rarely achievable, setbacks in implementation of financial incentives should be taken as an opportunity for revisions, redesign, and self-correction.

## Footnotes

1 The purposive selection was done to ensure that hospitals undergoing revitalisation were included, as it constituted the focus of the broader study.
